# Long-Term Vitamin D Deficiency Results in the Inhibition of Cell Proliferation and Alteration of Multiple Gastric Epithelial Cell Lineages in Mice

**DOI:** 10.3390/ijms23126684

**Published:** 2022-06-15

**Authors:** Shaima Sirajudeen, Iltaf Shah, Mohammed Akli Ayoub, Sherif M. Karam, Asma Al Menhali

**Affiliations:** 1 Department of Biology, College of Science, United Arab Emirates University (UAEU), Al Ain P.O. Box 15551, United Arab Emirates; 201890080@uaeu.ac.ae (S.S.); mayoub@uaeu.ac.ae (M.A.A.); 2 Zayed Centre for Scientific Research, United Arab Emirates University (UAEU), Al Ain P.O. Box 15551, United Arab Emirates; altafshah@uaeu.ac.ae (I.S.); skaram@uaeu.ac.ae (S.M.K.); 3 Department of Chemistry, College of Science, United Arab Emirates University (UAEU), Al Ain P.O. Box 15551, United Arab Emirates; 4 Department of Anatomy, College of Medicine and Health Sciences, United Arab Emirates University (UAEU), Al Ain P.O. Box 15551, United Arab Emirates

**Keywords:** VD, VD deficiency, Pdia3, nuclear VDR, darkness, diet, mucus cells, parietal cells, zymogenic cells, gastric mucosa, acid secretion

## Abstract

Over one billion people globally are vitamin D (VD) deficient. Studies on the biological roles of VD are numerous but very little on the stomach. This project aims to understand how gastric homeostasis is affected by VD deficiency caused by prolonged exposure to darkness alone or combined with VD deficient diet. Three groups of C57/BL6 mice were subjected to different light exposure conditions and diets for 12 months (*n* = 8–12/group): control—12 h/12 h light/dark ***SDL*** (***S***tandard ***D***iet/***L***ight), 24 h dark ***SDD*** (***S***tandard ***D***iet/***D***ark), and 24 h dark ***VDD*** (***VD*** deficient diet/***D***ark). Stomach samples were collected for different multi-label lectin-/immuno-histochemical and qRT-PCR analyses, and the serum for LC-MS-MS. We found that the membrane VD receptor is expressed widely in the stomach when compared to nuclear VD receptors. Compared to *SDL*, *VDD* mice developed mucous cell expansion with increased mucins-mRNA (3.27 ± 2.73 (*p* < 0.05)) increased apoptotic cells, 15 ± 7 (*p* ≤ 0.001)); decreased cell proliferation, 4 ± 4 (*p* < 0.05)) and decreased acid secretion 33 ± 2 μEq/kg (*p* ≤ 0.0001)). Interestingly, mice exposed to full darkness developed mild VD deficiency with higher VD epimer levels: 11.9 ± 2.08 ng/mL (*p* ≤ 0.0001)), expansion in zymogenic cell number (16 ± 3 (*p* ≤ 0.01)), and a reduction in acid secretion (18 ± 2 μEq/kg (*p* ≤ 0.0001)). In conclusion, changes in light exposure or VD levels have serious physiological effects on the gastric mucosa, which should be considered during the management of gastric disorders.

## 1. Introduction

Vitamin D (VD) is a major steroid hormone, and its deficiency has been associated with various health disorders, including bone diseases [1], adverse pregnancy effects [2], autoimmune [3], cardiovascular [4] and psychological disorders [5]. VD circulates in the body as inactive 25(OH)D, which is activated in a long process starting in the skin after exposure to solar UV-B radiation and passing through hydroxylation steps in the liver and kidneys to form the final active molecule of VD or 1,25(OH)_2_D. VD requires vitamin D receptors (VDRs) to exert their effects on biological systems. There are two types of receptors, the most widely known being nuclear VDR (nVDR) and the other being membrane VDR (mVDR), also known as Pdia3 (Protein disulfide isomerase family A member 3) [6,7]. Previous research from our lab has shown that nVDR and the cytochrome P450 (CYP450) group of enzymes required for VD activation are expressed in murine stomach tissues [8]. Recent discoveries about Pdia3 have unmasked interesting functions, but regardless of the growing interest in this area, Pdia3 is a highly understudied VD receptor [9,10].

The primary sources of VD are cutaneous exposure to sunlight (UV-B) and a balanced diet [11]. Sources of VD are easily accessible, but the rate of VD deficiency is alarmingly high. The percentage of the global population suffering from VD deficiency is increasing for various reasons. One reason is low exposure to the sun throughout the year due to little or no sun exposure during the winter and in hot places where people stay away from the sun. The dress style of different cultures is another important factor. Consuming an unbalanced diet with low levels of VD exacerbates the situation. Food items such as fish are the main sources of vitamin D, while the amount of VD in other products is considerably small, making its availability through the diet difficult [12]. Moreover, there are regions and/or populations with limited sources of vitamin D either due to high prices or scarcity of marine food products [13]. These situations can be overcome using fortified food and VD supplements, but they may be expensive or unaffordable for the common person, leading to becoming dependent on cheap but tasty food that has a poor VD content. The COVID-19 pandemic has also forced people to quarantine for long periods at home worldwide, preventing sunlight exposure [14,15].

For clinical screening of VD status, a total 25(OH)D level is currently the most common marker. However, there are other metabolites that may affect the VD level read-out accuracy. The 25(OH)D molecule is not the active form of VD. Its biological functions in the body are executed by the active form of VD called 1,25(OH)_2_D. Epimers are an interesting group of VD metabolites in that they play important roles in pregnancy and infants, while more roles are being discovered [16,17]. Numerous methods are used for the clinical assessment of VD levels, but the most accurate diagnostic tool is Liquid chromatography with tandem mass spectrometry (LC-MS-MS) [18]. LC-MS-MS is considered the ‘gold standard’ and the reference assay for the determination of VD levels because it measures both the active and inactive metabolites of VD. LC-MS-MS offers the advantages of being highly accurate and measuring several metabolites simultaneously and helps with the discovery of metabolites that are not so well studied [19].

The gastric gland in the corpus of mice has different cell lineages, namely pit, parietal, zymogenic, and enteroendocrine. These cell lineages are derived from multipotent stem cells located in the isthmus region of the glands [20,21,22]. Mucous-producing pit cells and mucous neck cells are responsible for secreting basic and acidic mucus, respectively [23]. Parietal cells are highly differentiated and secrete hydrochloric acid, which aids in the digestion of food and provides a chemical barrier against many pathogens. Highly coordinated and regulated actions of Cl^−^ and K^+^ channels/transporters and H^+^,K^+^-ATPase located on the membranes of cytoplasmic tubulovesicles lead to gastric acid secretion [24]. Upon stimulation, a complex membrane translocation process takes place in the parietal cells, which results in the localization of the H^+^,K^+^-ATPase on canalicular membranes and the pumping of H^+^ to the canalicular and gland lumen [22]. Mucous cells play a role in protecting the wall of the stomach by secreting a thick layer of mucus that clings to the luminal surface. Zymogenic cells are formed by the transdifferentiation of mucous neck cells [25]. The functions of parietal cells and zymogenic cells are intertwined. Zymogenic cells secrete pepsinogen C, which requires gastric acid for activation [25]. Tight regulation of the parietal cells, involving mechanisms guarded by the vagus nerve and numerous hormonal secretions such as gastrin, histamine, ghrelin, and somatostatin, ensures the proper secretion of gastric acid and the homeostasis of the gastric epithelium [22].

The aim of this paper is to investigate the different effects of moderate and severe VD deficiency on the mouse gastric glands, respectively, induced by prolonged exposure to darkness alone or combined with VD deficient diet. We hypothesized that VD plays a role in maintaining the homeostasis of the gastric gland along with preserving the acid-secreting machinery of the parietal cells.

## 2. Results

### 2.1. Creating VD Deficient Mouse Model

**Figure 1:**
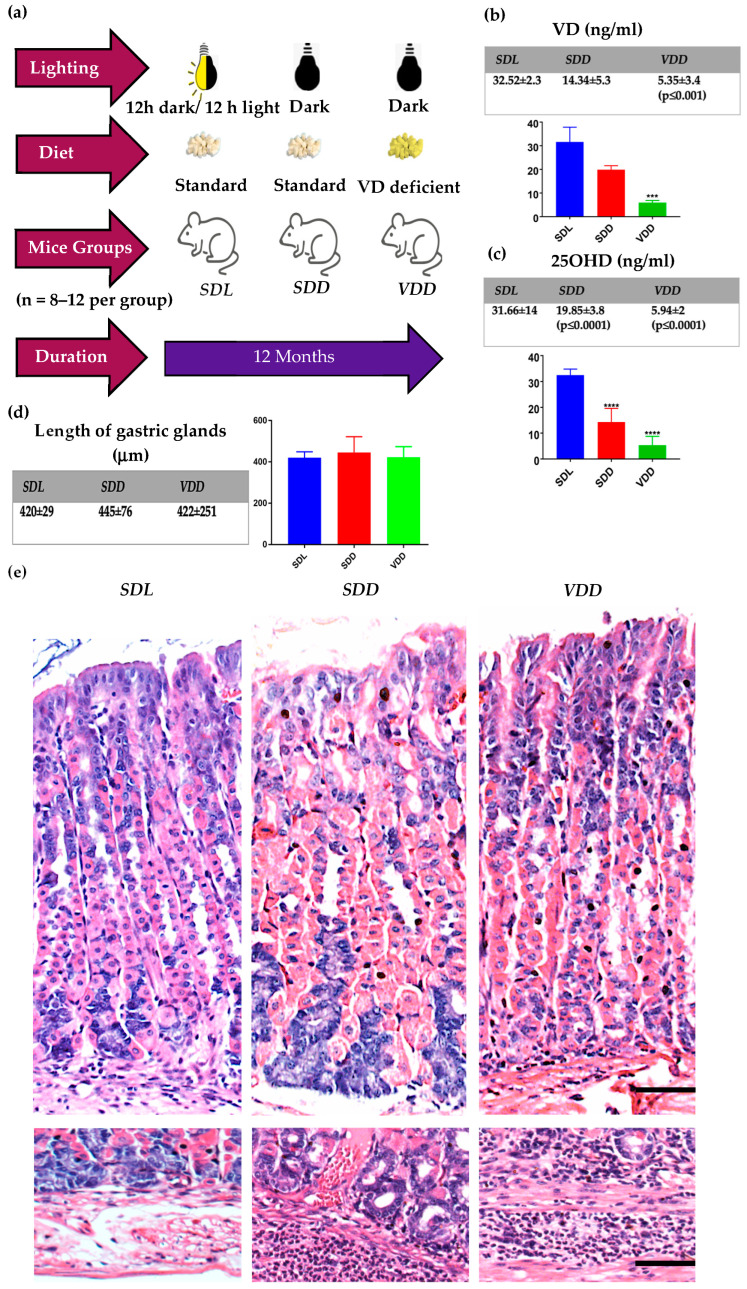
Experiment design. (**a**) Schematic representation of the experimental plan; (**b**) Total VD and (**c**) 25(OH)D serum levels as revealed by LC-MS-MS analysis; (**d**) Length of the gastric glands (μm), 5–7 animals per group (**e**) Hematoxylin and Eosin (H&E) stained sections of the corpora of the mice stomach. Insert—immune cells were seen to infiltrate the submucosal regions of *SDD* and *VDD* mice (scale bar = 200 μm). All mice were 12 months old, *n* = 8–12 per group, and statistics are presented as mean ± SD. One-way ANOVA was used for data analysis. *** *p* ≤ 0.001, **** *p* ≤ 0.0001.

To understand the difference between VD deficiency brought about by continuous exposure to darkness and/or due to dietary deficiency, we divided the mice into groups, as explained in Figure 1a.

By using LC-MS-MS, as expected, the serum levels of both total VD metabolites and 25(OH)D3 were significantly reduced in ***SDD*** and ***VDD*** compared with controls (*p* ≤ 0.001 and *p* ≤ 0.0001, respectively) (Figure 1b,c). Interestingly, serum 3-epi-25(OH)D3 levels were higher in ***SDD*** animals (*p* ≤ 0.0001), which shows that epimers and darkness may be related (Supplementary Figure S1).

***SDL*** mice had normal morphological features and were tolerant of the experimental conditions. Looking at the body weights, animals in the ***SDD*** and ***VDD*** groups exhibited changes in their body weights throughout the experimental period for unknown reasons (Supplementary Figure S2a,b)). In control females, the body weights were generally less than males, and there was no increase in body weight after week 32.

Also, when the female animals in ***SDD*** and ***VDD*** groups reached the last week of the experiment, they strikingly showed a more significant difference compared to the male groups. Thus, it seems that female mice are more sensitive to VD deficiency than males. To check whether the change in body weight is reflected in the body fat composition, we measured the gene expression of the fat marker adiponectin. Gene expression studies of adiponectin did not show significant changes when compared to controls (Supplementary Figure S2c).

To define the impact of diet- and darkness-induced VD deficiency, we investigated the morphological changes in the gastric epithelium. Although macroscopic examinations of ***SDL***, ***SDD*,** and ***VDD*** stomachs showed normal gastric mucosae with similar heights of the gastric glands, Figure 1d, microscopic examinations revealed glandular and cellular changes in ***SDD*** and ***VDD*** mice. Histological analysis of the gastric tissue sections of ***SDL*** mice indicated that the corpus and antrum had long and neatly packed gastric glands. Each gastric pit was clearly observed, and the borders between the cells were clear and well-organized. On the other hand, although parietal cells appeared to be normal among all three groups, it was obvious that the ***SDD*** group had more zymogenic cells compared to both ***SDL*** and ***VDD***. Dilatations in the gland lumen at the neck regions were prominent in ***VDD*** compared to ***SDL*** mice (Figure 1e). Moreover, the submucosa of ***SDD*** and ***VDD*** stomachs tended to have greater inflammatory cell infiltration compared to ***SDL***. In view of these observations, we conducted a detailed phenotypic analysis of the gastric mucosa of the mice. No sex-related histopathological differences were observed.

### 2.2. Reduction in Mucous Cells in SDD and Their Expansion in VDD Stomachs

**Figure 2:**
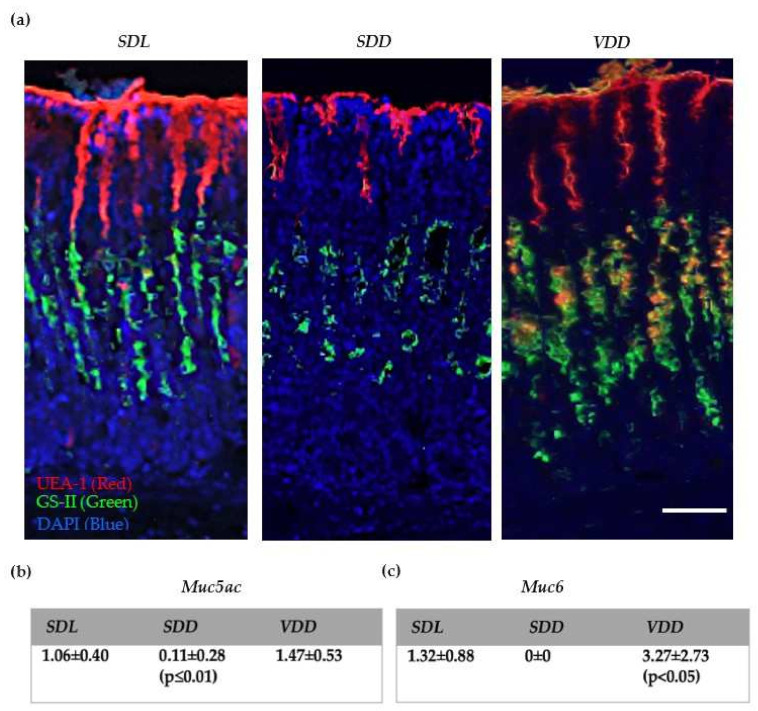
VD deficiency and mucous cell lineages. (**a**) Lectin histochemistry using UEA I and GS II binding assay on the gastric mucosa. Gene expression analysis (2^(−DDCt)) of (**b**) *Muc5AC* and (**c**) *Muc6* by qRT-PCR. The mice were 12 months old, *n* = 8–12 per group (scale bar = 200 μm). Data are presented as mean ± SD. One-way ANOVA was used for data analysis. *p* < 0.05 is considered statistically significant.

In order to study the distribution of mucous cells in a chronically VD-deficient stomach, paraffin sections were stained with both lectins UEA I and GS II that detect fucose and N-acetyl-D-glucosamine normally present in mucins of the surface mucous cells or mucous neck cells, respectively (Figure 2a). ***VDD*** mucosa exhibited an expansion of mucous neck cells. The co-expression of both carbohydrate residues in some of the neck cells was observed in ***VDD*** mice. The expansion of mucous neck cells correlated with the gene expression of *Muc6* (* *p* < 0.05), which is the marker for mucous neck cells (Figure 2a,c). Interestingly, ***SDD*** stomachs showed reduced lectin staining and a significant change of around 90% (** *p* ≤ 0.01) in surface mucus cell marker, *Muc5ac,* gene expression, compared to control (Figure 2a,b, Supplementary Figure S3).

### 2.3. Reduced Proliferation and High Apoptosis Rates in VDD Mice

**Figure 3:**
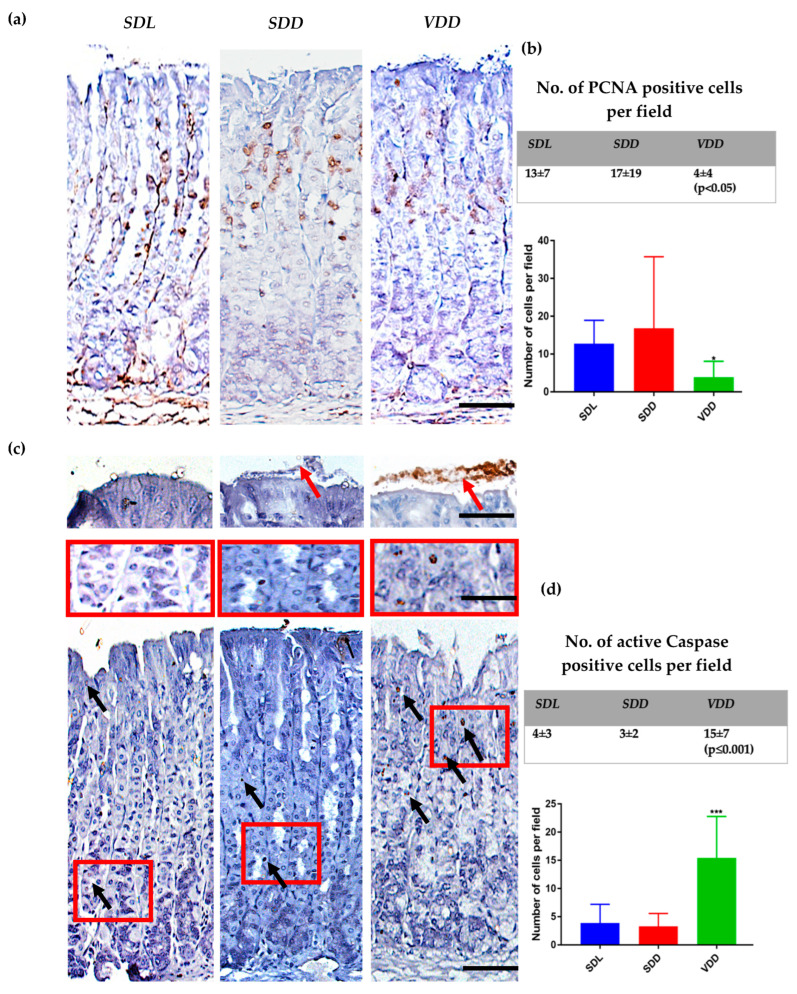
VD deficiency caused reduced proliferation and increased apoptosis rates. (**a**) Immunohistochemical analysis of proliferating cells using PCNA antibodies (scale bar = 200 μm); (**b**) Quantitative analysis of PCNA-positive cells. Kruskal–Wallis test was used for data analysis; (**c**) Immunohistochemical analysis of using activated caspase antibodies for apoptotic cells. Black arrows = cells undergoing apoptosis (scale bar = 200 μm). Insert = image of the parietal cells undergoing apoptosis. Red arrows = apoptotic cells being shed out of the mucosa (scale bar = 100 μm); (**d**) Quantitative analysis of apoptotic cells. The mice were 12 months old, *n* = 8–12 per group. Data are presented as mean ± SD. One-way ANOVA (Dunnett’s test) was used for data analysis. * indicates significant differences from the control group. * *p* < 0.05, *** *p* ≤ 0.001.

Immunohistochemical analysis of the gastric tissue sections was performed with anti-PCNA antibodies (Figure 3a), followed by counting the number of PCNA-positive proliferating cells per field. There was a significant decrease in the proliferation rate in ***VDD*** gastric glands when compared to ***SDL*** or ***SDD*** (Figure 3a,b).

On the other hand, apoptosis was studied by active caspase antibody staining, and there was a dramatic and significant increase in the number of cells undergoing apoptosis in ***VDD*** (Figure 3c,d). Although a 15-fold increase in active caspase positive cells was measured in cells found in the glandular epithelium, strands of unattached apoptotic cells were observed shedding out of gland pits, indicated by red arrows (Figure 3c).

### 2.4. Increased Zymogenic Cell Lineage in VD Deficient Corpus

**Figure 4:**
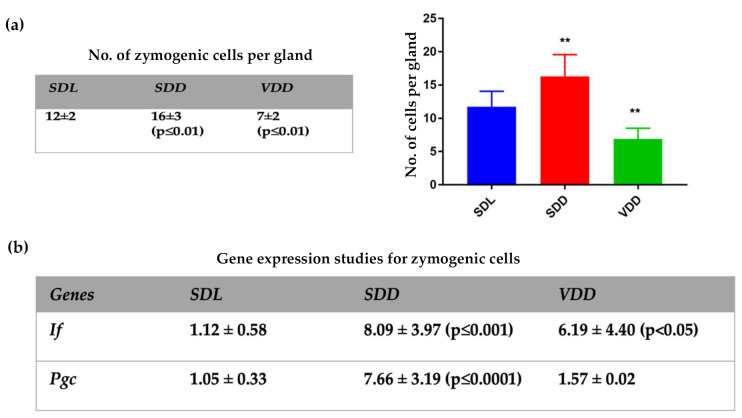
VD deficiency and zymogenic cells. (**a**) Quantitative analysis of zymogenic cells per gland. (**b**) Gene expression studies (2^(−DDCt)) for *IF* and *Pgc.* Data are presented as mean ± SD. One-way ANOVA was used for data analysis. The mice were 12 months old, *n* = 7–9 per group. *p* < 0.05 is considered statistically significant. ** *p* ≤ 0.01.

Mucous neck cells differentiate from pre-neck cells; subsequently, mucus neck cells transdifferentiate into fully functional zymogenic cells as they migrate toward the base of the gastric gland [20,26,27]. In order to investigate whether VD deficiency could bring about changes in this process, gene expression of zymogenic cell markers was analyzed. There were significant changes in the number of zymogenic cells (Figure 4a), with a significant increase in number in the ***SDD*** and a reduction in cell numbers in the ***VDD***group (Figure 4a). There was a significant increase in the intrinsic factor (*IF*) (Figure 4b and Supplementary Figure S4a) in the ***SDD*** and ***VDD*** groups compared to the control. However, pepsinogen C (*Pgc*) gene expression (Figure 4b and Supplementary Figure S4b) showed a significant increase (**** *p* ≤ 0.0001) in the ***SDD*** group only when compared to the control. These data matched our observations for the hematoxylin and eosin staining in Figure 1e.

### 2.5. Variable Endocrine Cell Lineages Response due to Chronic VD Deficiency

In order to study the changes that may take place due to VD deficiency on endocrine cells, first gastrin and the general endocrine marker chromogranin A (*Chga*) gene expression were quantified in all three groups (Table 1 and Supplementary Figure S5). The results of *Chga* showed variability in ***SDD*** and ***VDD*** readings, and as a result, there were no significant changes compared to ***SDL***, whereas the gastrin (*Gast*) gene showed a significant decrease in the ***VDD*** group compared to the control. In order to look at more endocrine cell-specific markers, Ghrelin hormone, *Ghrl,* and the histidine decarboxylase enzyme, *Hdc* gene expressions were analyzed for Ghrelin and ECL cells, respectively. *Ghrl* gene expression exhibited about a three-fold increase in ***SDD*** only when compared to ***SDL***. There were no significant changes in *Hdc* gene expression among the three groups. Since both ***SDD*** and ***VDD*** were in complete darkness throughout the study, we measured gene expressions of melatonin hormone receptors 1 and 2, *Mt1* and *Mt2*, and found a huge increase in the relative gene expression in ***SDD*** mice, reaching ~150- and ~200-fold, respectively, compared to ***SDL***. Interestingly, ***VDD*** and ***SDL*** showed similar expressions of melatonin receptors.

### 2.6. Impaired Function for Parietal Cells in SDD and VDD Groups

**Figure 5:**
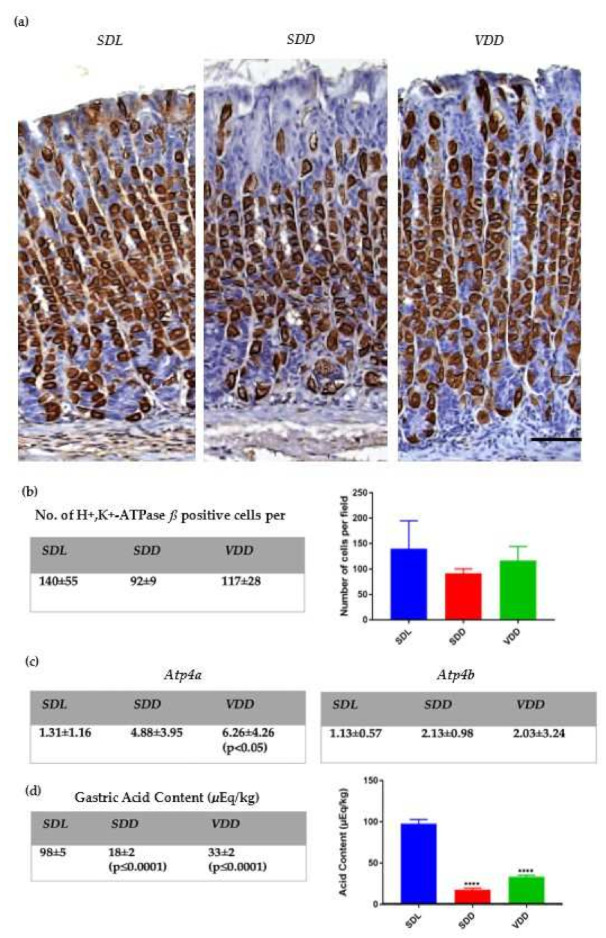
VD deficiency and parietal cell abnormal structure and function. (**a**) Gastric mucosal sections from ***SDL, SDD*,** and ***VDD*** mice were stained for H^+^,K^+^-ATPase ß-subunit using the antibody (scale bar = 500 μm); (**b**) Quantitation of H^+^,K^+^-ATPase ß-labeled cell counts per field (five glands per field); (**c**) Analysis of *Atp4a* and *Atp4b* in the mouse gastric tissues as revealed by qRT-PCR (2^(−DDCt)); (**d**) Acid content measurement. Data are presented as mean ± SD. All data were obtained from 12-month-old mice, *n* = 8–11 per group. *p* < 0.05 is considered statistically significant. **** *p* ≤ 0.0001.

In order to study whether parietal cell function would change under conditions of VD deficiency, parietal cells were immunoprobed with antibodies specific for the proton pump beta subunit. It was hard to distinguish any difference between the three groups (Figure 5a). Although quantitative analysis showed a slight decrease in parietal cell number in both ***SDD*** and ***VDD*** compared to the control (Figure 5b), this decline was insignificant. Surprisingly, mRNA expression of H^+^,K^+^-ATPase α (*Atp4a*) was increased in ***VDD*** compared to ***SDL***. No significant change was seen in the expression of H^+^,K^+^-ATPase ß (*Atp4b*) among the three groups (Figure 5c and Supplementary Figure S6). To connect the proton pump gene expression to the acid secretory function, gastric acid contents were measured for the three groups. There was around 80% and nearly 70% reduction in gastric acid content in ***SDD*** and ***VDD*** stomachs, respectively (Figure 5d).

### 2.7. Chronic VD Deficiency Causes Changes in VDR Expression with Induction or Repression of Respective Target Genes

**Figure 6:**
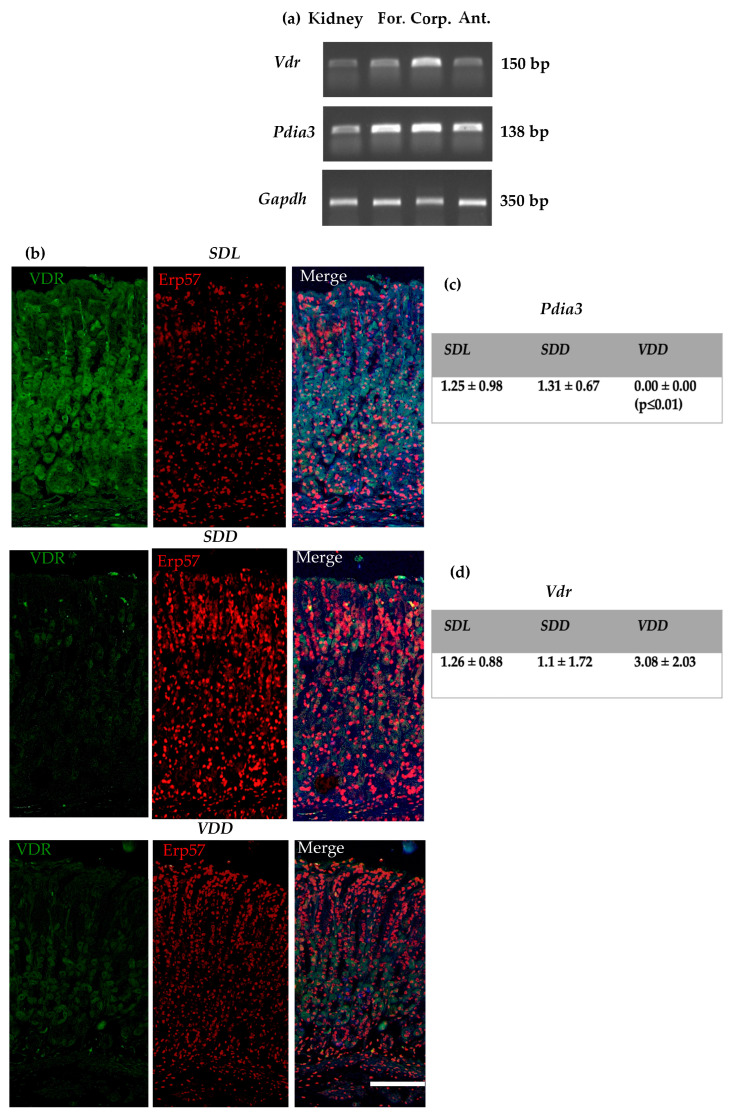
Distribution of VD receptors in the stomach (**a**) Conventional PCR for nVDR and Pdia3 in the wild type stomach (**b**) Immunohistochemical analysis for the three groups of mice using anti-nuclear VDR and anti-Erp57 antibodies (scale bar = 500 μm). (**c**) *Pdia3* and (**d**) *Vdr* gene expression levels (2^(−DDCt)) as determined by qRT-PCR and normalized to *Gapdh*. All mice were 12 months old, *n* = 8–12 per group. Data are presented as mean ± SD. One-way ANOVA (Dunnet’s test) was used for data analysis. *p* < 0.05 is considered statistically significant.

Cellular localization of nVDR and Pdia3 in the stomach of 12-month-old mice indicated that the receptors could be membranous/cytoplasmic/nuclear. We investigated whether the receptor distribution would change during the prolonged VD deficient treatment in mice. Conventional PCR results (Figure 6a) showed that Pdia3 has a holistic distribution compared to nVDR in the three parts of the stomach, namely, the forestomach, corpus, and antrum. *Pdia3* expression was significantly lower, whereas *Vdr* expression showed no significant change in the ***VDD*** group compared to the control. Immunohistochemistry results indicate that the expression of Pdia3 increased and nVDR decreased in the ***SDD*** group, but there were no significant changes in the gene expression analysis (Figure 6c,d and Supplementary Figure S7) when compared to the control.

VD exerts its function through the induction or suppression of target gene expression responses to Pdia3 and nVDR. In the current study, the following target genes (Table 2, Supplementary Figure S8) were investigated: caveolin (*Cav1*) and Src (*Src*) for Pdia3, and p21 (*P21*), Trpv6 (*Trpv6*), PTHLH (*Pthlh*) and PTHLH receptor (*Pthr1*) for nVDR. The expression of *Cav1* and *Src* showed no significant changes in the ***SDD*** and ***VDD*** groups. There was upregulation of *p21* in ***VDD*** mice and *Trpv6* and *Pthr1* in the ***SDD*** group.

## 3. Discussion

There are few studies that have investigated the role of VD in preserving the normal cellular make-up and function of the gastric mucosa [8,28,29]. In the current study, LC-MS-MS analyses of serum samples from the three groups of animals are conducted to confirm the VD levels, which revealed surprising results. Although ***SDD*** animals were provided with a standard diet, they were VD-deficient. This supports the fact that light is necessary for maintaining VD levels [6,15]. Moreover, light affected VD structure, and the levels of the epimer 3-Epi25(OH)D were high in the ***SDD*** group. It is known from the literature that embryos, newborns, and infants, as well as their pregnant mothers, have higher levels of epimers in their bodies [30]. This is exciting because the infants start their life in the darkness of the womb, which could contribute to the rise in epimer 3-Epi25(OH)D [31]. It is interesting that we observed more than a 12-fold rise in the epimer 3-Epi25(OH)D. However, the way in which epimers affect the body and its relationship to darkness is not fully understood.

There are many studies that connect VD with obesity or weight loss. In our study, we screened the animals every week for one year and noticed this conflict in weight gain/loss throughout the experimental period. However, this variation in body weight agrees with previous research, suggesting that VD deficiency may or may not be a contributing factor to weight gain [32,33] and loss [34,35,36]. In these studies, the bodyweight measurement may have been conducted over short time periods while the samples were going through this turbulent phase of weight gain or loss. The animals were housed separately based on gender, and we first attempted to correlate body weight with gender, but we failed to reach any conclusions because both genders exhibited the same trend in weight gain and loss. To assess whether appetite plays a role in weight gain, we checked the gene expression of the Ghrelin hormone. The animals in the ***SDD*** and ***VDD*** groups were obese, but only the ***SDD*** group had significantly higher levels of ghrelin. This finding might indicate a correlation between darkness and ghrelin levels. We tried to connect weight with adiponectin levels but did not notice any significant change. There may be a system in the body for overcoming VD deficiency resulting in the instability of body weight. Additionally, week 24 raises more questions because this is where huge changes happened. It would be exciting to study the reasons behind these phenomena by having mice of the three groups at this timepoint and correlate body weight with parameters such as gene expression levels of adiponectin and ghrelin.

Another striking finding is that there were changes in mucus cell lineages in the ***SDD*** and ***VDD*** groups. Normally, surface mucous cells are found on the pit and mucous neck cells are found deep in the middle of the gastric glands. They secrete Muc5AC and Muc6 mucins, respectively [37]. The descending mucus neck cells transdifferentiate into zymogenic cells [38,39]. It seems that in the dark, the mucus neck cell lineage undergoes enhanced transdifferentiation to form zymogenic cells. This is supported by an increase in the zymogenic cell numbers in the ***SDD*** group. On the other hand, when animals were in the dark and consuming a VD-deficient diet, there was higher expression of mucus cell marker, Muc6, which may be due to the increased production and differentiation of mucous neck cells (Figure 2). The expansion of mucous cells affected gastric gland compartments, where we see the co-existence of Muc5AC and Muc6 in different areas, which are visible as yellow to orange-colored regions. This could be due to the activation of stem cells and the expansion of the mucous cells. The development of mucous cells with dual features of pit and neck regions supports the fact that they have a common source of origin [20,39]. Correspondingly, abnormalities in the differentiation of ***SDD*** gastric mucosa were seen in the zymogenic cells, which was reflected in the cell number and gene expression analyses.

One of the interesting phenotypes is the one related to parietal cell function and viability. There were discrepancies in the expression of proton pumps due to variability between the samples and a significant decrease in acid secretion in ***SDD*** and ***VDD*** mice compared to the control. Histamine, gastrin, and acetylcholine are known to be key secretagogues of gastric acid. HDC is the key enzyme necessary for the generation of histamine [22]. There was a significant reduction in gastrin levels, while a change in HDC was insignificant due to the variability between the samples in the ***VDD*** mice compared to the control. A possible explanation for the low acid secretion could be that in the absence of VD, the transportation proteins/structures may be defective, but more studies are required to prove this. Despite all of this, the total number of parietal cells seemed to be maintained by morphometric analysis. It would be interesting to examine the changes at the ultrastructural level using transmission electron microscopy as well as look into the effects of VD deficiency in animals at earlier stages of deficiency.

Previous studies have suggested that VD deficiency can cause reduced cell proliferation [40,41] and apoptotic cell death [42,43]. In the ***VDD*** group, there was a significantly lower rate of proliferation and a higher rate of cell death, where apoptotic cells were seen to be extruded from the gastric mucosa. Our findings complement previous studies in this respect, where VD deficiency was seen to inhibit cell proliferation and increase apoptosis [44,45,46]. It would be interesting to study the pathways associated with apoptosis in VD deficiency and to describe the possible mechanism behind it.

There are two types of receptors for VD—the nuclear VDR and Pdia3. Nuclear VDR is a type of nuclear hormone receptor, and most of the biological actions are mediated through the VDR-mediated control of target genes [47]. Pdia3 is a pleiotropic, ER-resident protein that has been detected on the cell surface [48], in the cytoplasm [49], and in mitochondria [50]. Pdia3 is located in cellular compartments other than the ER and is involved in a variety of cellular functions such as signal transduction, protein trafficking, interacting with mTOR, and regulation of STAT3 signaling [51,52]. In the absence of light or diet supplemented with sufficient VD, we see differential expressions of receptors, which was surprising due to the low VD levels. Our results do not complement previous findings, which indicated that VD had a significant correlation with the expression of nuclear VDR [53,54]. This shows that the nature of their expression changes depending on the nature of the diet and light exposure, and the two receptors may take turns carrying out their functions. The link between darkness and the mode of action of the receptors would be an interesting area of research.

The receptors have different mechanisms of action, and it would be interesting to look at a few of the downstream target genes. Caveolin and Src are proteins that work in association with both Pdia3 and VDR. Src helps with the rapid movement of Ca^2+^ across the cell membrane in the presence of VD and is a target of Pdia3 and occasionally works in association with VDR [55]. The gene expression of *Cav1* and *Src* did not show significant changes in the ***SDD*** and ***VDD*** groups. While nVDR gene expression did not show significant changes in ***SDD*** mice, there was high expression of *Pth1r* and *Trpv6* (target genes of VDR), ~30-fold and ~10-fold, respectively, but in ***VDD***, the change was insignificant. Previous studies have shown that VDR has the ability to suppress the expression of genes that are induced in the presence of ligands, and thus, the phenomenon noticed in our studies was not in accordance with previous literature [56]. The p21 gene is involved in tumorigenesis and acts both as a tumor suppressor and an oncogene, depending on the cellular environment [44,57]. In the ***VDD*** group, the relative fold gene expression of p21 was also significantly higher (~50,000-fold). Thus, the expression of the downstream target genes is possibly regulated by several factors, such as the availability of the ligand (VD) or the expression of Pdia3 and nVDR.

Although the goal of this study was to investigate the effects of VD deficiency caused by darkness and a VD-poor diet, our ***SDD*** group, which is an important control group for the experiment, surprisingly yielded remarkable results. ***SDD*** was not a major experimental group in this study. However, high ghrelin expression, a decrease in mucous cell lineages, increased zymogenic cells and high levels of epimers were the most striking features in the ***SDD*** animals. Due to the exciting findings obtained in the chronic absence of light, it was necessary to check whether melatonin, the darkness hormone, had an impact on these changes. Melatonin acts through the melatonin receptors (MT1 and MT2) and mediates the dark signals and stabilizes the circadian rhythms in the body. In the gastrointestinal tract, it is synthesized by the EC cells [58,59], under the influence of a variety of stimuli such as nutritional factors, and the gene expression of *MT1* and *MT2* was seen to be higher in the ***SDD*** group. Actin [60], ezrin [61], and Huntington interacting protein 1-related (Hip1r) are a few of the cytoskeletal proteins responsible for the transformation of the tubulovesicular system, microvilli, and canaliculi, as well as vesicular transport in the parietal cells. Major structural changes in the parietal cells and low acid secretion were seen in the VD deficient groups. VD thus likely plays a major role in the formation of cytoskeletal proteins and assists in the secretion of gastric acid into the lumen and would make an interesting area of study.

There are several limitations to this study. There is a need to investigate the ultrastructural changes and the mechanisms involved in the changes observed in the gastric stem/progenitor cells and the different gastric epithelial cell lineages. It would also be important to study a group of animals that are provided a vitamin D deficient diet and exposed to normal light. The latter would confirm and also differentiate between the findings of exposure to dark versus vitamin D deficient diet alone and, therefore, add an additional dimension to this study.

## 4. Materials and Methods

### 4.1. Experimental Animals

This study was conducted with the approval of the Animal Research Ethics Committee (A-REC) of the United Arab Emirates University (Protocol# ERA_2017_5684).

### 4.2. Mice and Diets

Three-week-old C57BL/6J mice were housed in a specific-pathogen-free animal facility at the College of Medicine and Health Sciences (CMHS) in UAEU. The mice were maintained in autoclaved filter-top cages under specific pathogen-free conditions with environmentally controlled temperature (23 ± 1 °C) and relative humidity (50 ± 10%) and were handled in a laminar flow hood. The weaned mice (*n* = 36) were divided into three equal groups based on light exposure and diet and maintained for 12 months. The control ***S***tandard ***D***iet ***L***ight **(*SDL*)** group was maintained on a standard AIN-93G rodent diet, which included 1000 IU VD3 (D10012Gi, Research Diet, New Brunswick, NJ, USA), with a 12 h light/12 h dark cycle. The second ***S***tandard ***D***iet ***D***ark **(*SDD*)** group was fed the standard diet and maintained in the dark with no overhead light. The third ***V***itamin **D** deficient ***D***ark **(*VDD*)** group was maintained in the dark and fed upon a specially formulated VD-deficient diet with a VD level of 25 IU/kg, D17053003 (Research Diet, New Brunswick, NJ, USA) [62]. Body weights were measured every week, and statistical analyses were conducted. At the end of 12 months, the mice were fasted overnight with free access to water prior to sacrifice. After euthanization, blood samples were collected, and the serum was separated and stored at −80 °C until analysis. The stomach was collected, cut along the greater curvature, divided, and prepared for various experiments. Part of the stomach was frozen immediately in liquid nitrogen (gene expression studies) or fixed in Bouin’s (IHC) or Carnoy’s (TEM studies) fixative.

### 4.3. Histology and Immunohistochemistry

To answer our research question, we used lectin histochemistry, immunohistochemistry (IHC), quantitative RT-PCR, and LC-MS-MS studies. Gastric tissues were collected and fixed in Bouin’s fixative. Paraffin sections (4 μm) were stained with hematoxylin and eosin (H&E) (H&E Staining Kit, ab245880, Abcam, Cambridge, UK) for the evaluation of general histology. The slides were deparaffinized, rehydrated, and incubated with hematoxylin and eosin for 5 and 3 min, respectively.

Immunohistochemistry for lectins was performed using lectin II GS-II (*Griffonia simplicifolia),* Alexa Flour^TM^ 488-conjugate (1 in 1000; L21415, Invitrogen, Thermo Fisher Scientific, Life Technologies, Carlsbad, CA, USA) and UEA I, Rhodamine (Ulex Europaeus Agglutinin, I, 1 in 1000; RL-1062-2, Vector Laboratories, San Fransisco, CA, USA) on surface and mucous neck cells, respectively. Parietal cells were identified by immunostaining for Anti-Proton Pump (H,K-ATPase β subunit) mouse monoclonal antibody (1 in 5000; D032-3, Medical and Biological Laboratories, Tokyo, Japan), and nVDR and Pdia3 were detected using VD3 receptor antibody (D2K6W) rabbit mAB (1 in 12,000, Cell Signaling Technology, Danvers, MA, USA) and ERp57 antibody (4E69, 1:200; sc-71075, Santa Cruz Biotechnology, Santa Cruz, CA, USA), respectively. The sections were briefly incubated with 1% bovine serum albumin (blocking reagent) for 1 h, and the primary antibodies were added and incubated overnight at 4 °C. Biotin-SP conjugated goat anti-rat IgG (H + L) antibodies (1:1000; 112-065-003, Jackson Immunoresearch, West Grove, PA, USA) and goat anti-rabbit IgG H&L (TRITC) (1 in 200; ab6718, Abcam, Cambridge, UK) antibodies were then added and incubated at room temperature for 1 h. The sections were then counterstained with hematoxylin and mounted with DPX. Imaging was carried out using an Olympus microscope IX83 and Cellsens software (Tokyo, Japan).

For co-immunofluorescence staining, antigen retrieval was performed using citrate buffer at 96 °C for 10 min. Tissue sections were then blocked with 1% bovine serum albumin for 1 h and incubated for 1 h at room temperature with ERp57 and VDR primary antibodies. The sections were then washed with PBS and treated with Cy3- or Alexa Fluor 448-conjugated goat anti-rat secondary antibodies (1 in 100; 112-165-003 or 112-545-003, Jackson ImmunoResearch) at room temperature for 1 h. The sections were washed with PBS and incubated with lectins specific to surface mucous cells (UEA I) or mucous neck cells (Lectin GS-II). We used 4′,6-diamidino-2-phenylindole (DAPI, ab104139, Abcam, Cambridge, UK) as a nuclear counterstain as well as for mounting the preparations. Negative controls for each experiment excluded primary antibodies.

### 4.4. Analysis of Proliferation and Apoptosis

Proliferating cells were identified in paraffin sections by immunostaining for PCNA. PCNA-positive cells were identified using a PC10 Mouse mAb (2586, 1 in 12,000; Cell Signaling Technology, Danvers, MA, USA). Biotinylated anti-mouse antibody (1 in 50; Invitrogen, Thermo Fisher Scientific, Life Technologies, Carlsbad, CA, USA) was used as secondary antibody. Apoptosis was studied by detecting the cleaved caspase using SignalStain Apoptosis Cleaved Caspase-3 IHC Detection Kit # 12692 (Cell Signaling Technology, Danvers, MA, USA) according to the manufacturer’s instructions. The results were visualized using Cellsens software and an Olympus microscope IX83 (Tokyo, Japan).

### 4.5. Morphometric Analysis

Cellsens software and an Olympus microscope IX83 (Tokyo, Japan) were used to measure the height of glands, and count the number of zymogenic cells, H^+^-K^+^-ATPase ß-subunits-, PCNA- and cleaved caspase-3 -positive cells. The data obtained were expressed as the number of positive cells per field/gland as required.

### 4.6. RNA Extraction

Total RNA was extracted from the corpus of the mouse stomach separately using TRIzol reagent. RNA purification was performed using an RNase-Free DNase kit (Qiagen). Individual stomach (~50–100 mg) tissues were first briefly homogenized in TRIzol Reagent (15596026-018) provided by Invitrogen (Thermo Fisher Scientific, Waltham, MA, USA) using a Bio-GenSeries PRO200 homogenizer (Oxford, MS, USA) and incubated at room temperature for 5 min for complete dissociation of the nucleoprotein complex. Tissue lysates were then incubated with chloroform for 3 min and centrifuged at ≥12,000× *g* for 15 min at 4 °C, and the aqueous phase containing the RNA was transferred to a fresh tube. A total of 0.5 mL of isopropanol was added to the aqueous phase and centrifuged for 10 min at 12,000× *g* at 4 °C. Total RNA precipitate, as a white, gel-like pellet, was resuspended in 75% ethanol. The pellet was washed, air-dried, and resuspended in 30 µL of nuclease-free water and incubated at 55–60 °C for 10–15 min. RNA samples were then subjected to DNase treatment using Qiagen RNase-free DNase set (Ref. No. 79254, Hilden, Germany). RNA samples were transferred to the spin column, and DNase was directly added and incubated for 15 min. This was followed by a series of wash steps, and the RNA stuck to the column was eluted out in 30 µL of nuclease-free water. The purity of the RNA samples was checked using a Thermo Scientific NanoDrop 2000 Spectrophotometer and agarose gel electrophoresis before proceeding to downstream applications or being stored at −80 °C.

### 4.7. cDNA Synthesis

RNA samples were standardized, and 1 μg RNA was reverse transcribed to cDNA using the iScriptTM cDNA synthesis kit (Bio-Rad, Hercules, CA, USA) according to the manufacturer’s instructions.

### 4.8. Gene Expression

To examine the expression patterns of *nVDR* and *Pdia3* in normal mouse gastric tissue, a PCR was conducted with cDNA using the GoTaq Flexi DNA polymerase kit (Promega, Madison, WI, USA) according to the manufacturer’s instructions. *GAPDH* was used as the internal control. PCR products were separated by gel electrophoresis in 1.5% agarose with ethidium bromide and visualized with a Gel DocTM EZ Imager (Bio-Rad, Hercules, CA, USA).

### 4.9. Quantitative Real-Time PCR

qRT-PCR was used for the differential expression of genes specific to Gapdh [63], gastric epithelial cell lineages [64,65,66,67], cell proliferation [68], and VDR [8], Pdia3/VDR direct target genes. RT-PCR reactions were performed on the cDNA using PowerUp SYBR Green Master Mix (A25742, Applied Biosystems, Thermo Fisher Scientific, Carlsbad, CA, USA) in a QuantiStudio^®^ 5 Real-Time PCR instrument (Applied Biosystems, Thermo Fisher Scientific, Foster City, CA, USA) according to the manufacturer’s instructions. cDNA was amplified, and quantification of the genes in the mRNA of each sample was performed in triplicates. All the primers used are presented in Appendix A Table A1. Gene expression levels were calculated using the comparative cycle threshold method (ΔΔCT method) to calculate relative fold gene expression 2^(−DDCt). The results were normalized to *Gapdh*.

### 4.10. Determination of Serum VD Levels by LC-MS-MS

Serum VD levels were measured via LC-MS-MS (liquid chromatography–mass spectrometry). Blood was collected from the posterior vena cava in terminally anesthetized animals. The animals were anesthetized, a ‘Y’-shaped cut was made in the abdomen, and the intestines were gently removed. The liver was pushed forward, and the posterior vena cava (between the kidneys) was identified. Blood was collected from the posterior vena cava using a syringe. This procedure was repeated three to four times to collect the maximum volume of blood possible. Approximately 200 μL–1 mL of blood was collected in 2 mL Eppendorf tubes. Serum was separated by centrifugation at 12,000× *g* (14,000 rpm) for 15 min at 4 °C, then stored at −80 °C. Liquid chromatography-tandem mass spectrometry (LC-MS-MS) was performed to quantify VD and its epimers [8].

The LC-MS-MS system consisted of an LC part, Nexera ultra-high-pressure liquid chromatography (UHPLC) system Nexera X2, (Shimadzu, Japan); connected to an MS/MS part, tandem mass spectrometer, model 8060 (Shimadzu, Japan), The UHPLC system had a degasser, auto-sampler, binary pump, and column heater. The mass spectrometer was used in positive electrospray ionization (ESI) mode causing a protonation to a molecular ion [M + H]+. In order to achieve the maximum sensitivity, the optimum MRM parameters were fixed for an individual analyte by the direct infusion of individual analytes of the standard compound. The mass spectrometer’s operating parameters for nebulization, drying, heating gas flows and interface, and heating block temperatures were optimized to achieve the best detection and quantification of the VD metabolites (Table 3). The nebulizing gas flow was set to 2 L/min, the drying gas flow was 8 L/min, the heating gas flow was optimized at 8 L/min, the interface temperature was kept at 300 °C, and the heating block temperature was set to 400 °C. The Lab-Solutions software (Shimadzu, Japan) was used for data generation and reporting. The different metabolites were separated using an Ascentis Express F5 column with a particle size of 2.7 µm and dimensions (150 mm × 2.1 mm), fitted with a pre-column guard. Mobile phase A consisted of aqueous 5 mM ammonium formate, while mobile phase B consisted of methanol with 5 mM ammonium formate. Gradient elution was used for sample detection and separation. The flow rate was kept at 0.5 mL/min.

Eight VD metabolites were investigated in serum, namely, 1,25(OH)_2_D (1,25(OH)_2_D2, and 1,25(OH)_2_D3)- 25(OH)D (25(OH)D3 + 25(OH)D2), including their epimer, 3-epi-25(OH)D (3-epi-25(OH)D3 and 3-epi-25(OH)D2), vitamin D (vitamin D3 + vitamin D2), and a bile acid precursor 7alpha-hydroxy-4-cholesten-3-one (7αC4), which is known to cause interference in LC-MS-MS analysis, and 25-hydroxyvitamin-D3(6,19,19-d3) was used as internal standard. The LC-MS-MS method was validated according to FDA-US guidelines [69]. The reported linear range for all the analytes was 0.5–150 ng/mL, except for 1,25(OH)_2_D, where the linear range was found to be 10 to 1000 pg/mL. The lower limit of quantitation (LOQ) for all the analytes was 0.5 ng/mL except for 1,25(OH)_2_D, for which the LOQ was 10 pg/mL. The lower limit of detection (LOD) was 0.29 ng/mL for all the analytes except 1,25(OH)_2_D, for which the LOD was 6 pg/mL. The selected MRM transitions, along with their respective parameters, are provided in Table 1.

### 4.11. Gastric Content pH Measurement

Stomach tissues were harvested from the three groups of mice. They were cut along the greater curvature and rinsed with 0.5 mL of 0.9% NaCl (pH 7.0). The contents were collected and centrifuged at 1848× *g* (5000 rpm) for 10 min. The supernatant was collected, and the acid content was quantified by titrations against 0.005 M NaOH as described elsewhere [70]. The values were normalized to the body weights, and the acid equivalent per kilogram of body weight (Eq/Kg) was calculated.

### 4.12. Statistical Analysis

GraphPad Prism 7.03 (GraphPad, Inc., San Diego, CA, USA) was used for the statistical analysis. Most of the RT-PCR data passed the DÁgostino and Pearson test for normality of distribution. All the data were expressed as means ± SD for real-time PCR, morphometric analyses, and VD metabolites. One-way or two-way Analysis of Variance (ANOVA), followed by Dunnett’s multiple comparison post-test, was used. The only non-parametric data presented were in the form of median for the morphometric analysis of the PCNA-positive cells, Figure 3b. In this case, the Kruskal–Wallis test was used. The Brown–Forsythe test and Bartlett’s test were used to test for the equality of group variance. * *p* < 0.05 was considered statistically significant.

## 5. Conclusions

From this study, it is evident that long-term exposure to darkness or a VD insufficient diet caused major physiological changes in the body of the animals. VD deficiency resulted in an increase in the levels of epimers, enhancement of the zymogenic cell lineage, increased apoptosis and decreased proliferation, and a reduction in acid secretion. This demonstrates that VD is necessary for preserving the acid-secreting machinery and overall cellular make-up of the gastric mucosa. It is amazing how changes in light exposure or VD levels have profound effects on the gastric mucosa, which can be thought of during the diagnosis and treatment of gastric disorders.

## Figures and Tables

**Table 1:** VD deficiency affects the gene expression of hormones.

Genes Studied	2^(−DDCt) ± SD		
	*SDL*	*SDD*	*VDD*
*Chga*	1.49 ± 1.10	1.42 ± 2.2	2.28 ± 2.02
*Gast*	1.47 ± 1.47	1.55 ± 2.06	0.22 ± 0.81 (*p* < 0.05)
*Ghrl*	0.95 ± 0.40	2.58 ± 1.02 (*p* ≤ 0.01)	0.44 ± 0.36
*Hdc*	1.16 ± 0.74	3.79 ± 8.144	0.18 ± 0.17
*Mt1*	1.53 ± 1.66	156.25 ± 240.62 (*p* < 0.05)	13.21 ± 22.20
*Mt2*	2.33 ± 4.17	195.64 ± 304.58 (*p* < 0.05)	1.9 ± 2.02

All mice were 12 months old, *n* = 7–12 per group. Results are presented as mean ± SD. One-way ANOVA was used for data analysis. *p* < 0.05 is considered statistically significant.

**Table 2:** nVDR and Pdia3 target gene expression in VD deficient mice.

Genes Studied	2^(−DDCt) ± SD		
	*SDL*	*SDD*	*VDD*
*Cav1*	2.37 ± 4.5	4.23 ± 5.17	3.76 ± 8.55
*Src*	1.05 ± 0.43	0.76 ± 0.6	0.56 ± 0.54
*p21*	7.23 ± 13.40	560.72 ± 994.18	56,925.58 ± 26,196.44 (*p* ≤ 0.0001)
*Trpv6*	1.47 ± 1.56	26.21 ± 9.12 (*p* ≤ 0.0001)	6.78 ± 1.76
*Pthlh*	1.84 ± 1.48	20.16 ± 25.65	0.32 ± 0.39
*Pthr1*	1.09 ± 0.46	8.74 ± 7.06 (*p* ≤ 0.01)	3.12 ± 1.56

All mice were 12 months old, *n* = 8–11 per group. Results are presented as mean ± SD. One-way ANOVA was used for data analysis. *p* < 0.05 is considered statistically significant.

**Table 3:** VD and its metabolites are shown along with respective precursor/product ions, retention times and collision energy. Transitions marked with asterisks (*) are quantifier ions and were used for quantitation.

No.	Analytes	Retention Time (min)	Precursor	Product	Collision Energy (eV)
			(m/z)	(m/z)	
1	1,25(OH)_2_D3	3.82	399.1	381.3 *	−14
2	1,25(OH)_2_D2	3.948	411.1	135.3 *	−13
				133.1	−12
3	25(OH)D3	6.98	383.2	365.3 *	−15
				107.1	−30
4	25(OH)D2	7.76	395.1	377.3 *	−17
				81.1	−38
5	3-epi-25(OH)D3	7.69	383.2	365.3 *	−15
				107.1	−30
6	3-epi-25(OH)D2	8.52	395.1	377.1 *	−17
				81.1	−38
7	Vitamin-D3	15.104	385	367 *	−13
				259	−16
				91	−56
8	Vitamin-D2	15.072	397.1	379.4 *	−17
				69	−19
9	7αC4	14.48	401.5	383.25	−16
				97.1 *	−29
				91.2	−23
10	[25 hydroxyvitamin-D3 (6,19,19-d3)]	6.98	386.3	368.2	−15
				257.2 *	−183
				95.2	−35

## Data Availability

The raw data used to generate the graphs are available upon request.

## Supplementary Materials

The following supporting information can be downloaded at: https://www.mdpi.com/article/10.3390/ijms23126684/s1.

## Appendix A

**Table A1:** Primer sequences.

Genes	Primer Sequence (5′->3′)		Product Size (bp)
*Gapdh*	TCAAGAAGGTGGTGAAGCAGG	TATTATGGGGGTCTGGGATGG	350
*Muc5ac*	CTGGTTTGACACTGACTTCCC	CTCCTCTCGGTGACAGAGTCT	103
*Muc6*	AGCCCACATTCCCTATCAGC	CACAGTGGAAGATTGCGAGAG	192
*Atp4a*	GCCTGTCACTGACAGCAAAGAGG	GGTCTTCTGTGGTGTCCGCC	189
*Atp4b*	AACAGAATTGTCAAGTTCCTC	AGACTGAAGGTGCCATTG	140
*Chga*	TCAAATGCCCTATCCAAGTCC	GCCAACCGTACTTCAAACTTCG	134
*Pgc*	TGCAAGGCATTGTAGACACA	CTCCTATGGTCTGCAGAAGTTCATT	81
*Gast*	CATCATCTGGACCAGGGACCAA	CCTCCATTCGTGGCCTCTGCT	150
*Ghrl*	GGCAGGCTCCAGCTTCCT	CTGGTGGCTTCTTGGATTCC	71
*IF*	CTTGGCCCTGACCTGTATGT	TAGGTTGCTCAGGTGTCACG	191
*Mt1*	GGGCCCCACTCAACCTCATAG	AGCAGTAAGACCCCAACCAGTGT	352
*Mt2*	AACCGCTACTGCTGCATCTGTCATGCCCAGGTCCAGTGGAG	AAACTGCGCAAATCACTCGGTCTC	341
*Tff2*		GAACTTTCTTCTGGCTTGCAG	468
*Pdia3*	ACTGGAGAGGTTCCTGTTGTGG	CAGGTATCTCTTCAAGTTGCCATC	138
*Vdr*	GAATGTGCCTCGGATCTGTG	ATGCGGCAATCTCCATTGAAG	150
*Cav1*	GACCCCAAGCATCTCAACGA	GCCATAGGGATGCCGAAGA	173
*Mapk*	CGAAATGACCGGCTACGTGG	CACTTCATCGTAGGTCAGGC	505
*Src*	TCCACACCTCTCCGAAGCAA	CATGCTGATGGCCTGTGTCA	151
*Pthlh*	AATGCATTGGGATCAAACTGTCT	GCCTTGGCAAAAGGGAAAA	75
*Pth1r*	GCTTCCTGCCACCACCAAT	GGGAATGTCATAGTAACTGG	102
*Trpv6*	ATCGATGGCCCTGCGAACT	CAGAGTAGAGGCCATCTTGTTGCTG	358
*P21*	GCCTTAGCCCTCACTCTGTG	AGCTGGCCTTAGAGGTGACA	431
*Lgr5*	TCTTCACCTCCTACCTGGACCT	GGCGTAGTCTGCTATGTGGTGT	419
*Nanog*	CCTGATTCTTCTACCAGTCCA	GGCCTGAGAGAACACAGTCC	123
*Adipoq*	CCTGGAGAGAAGGGAGAGAAA	CGAATGGGTACATTGGGAAC	209
