# Supplementary material for: Long-Term Vitamin D Deficiency Results in the Inhibition of Cell Proliferation and Alteration of Multiple Gastric Epithelial Cell Lineages in Mice
Source: Int J Mol Sci. 2022 Jun 15;23(12):6684. doi: 10.3390/ijms23126684 (PMC9224370; doi:10.3390/ijms23126684)
Supplement: Supplementary file 1 [file ijms-23-06684-s001.zip › ijms-1718625-supplementary.pdf]

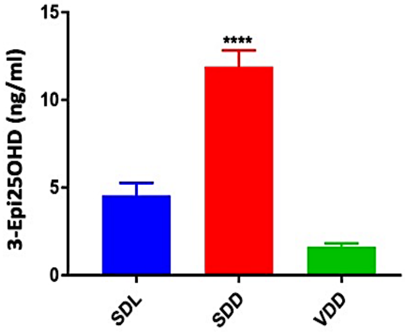

Figure S1. Levels of serum VD epimers revealed by LC-MS-MS. All mice were 12 months old, n = 8–12 per group. Data are presented as mean ± SE. One-way ANOVA was used for data analysis. * indicates significant differences from the control group. **** $p \leq 0.0001$.

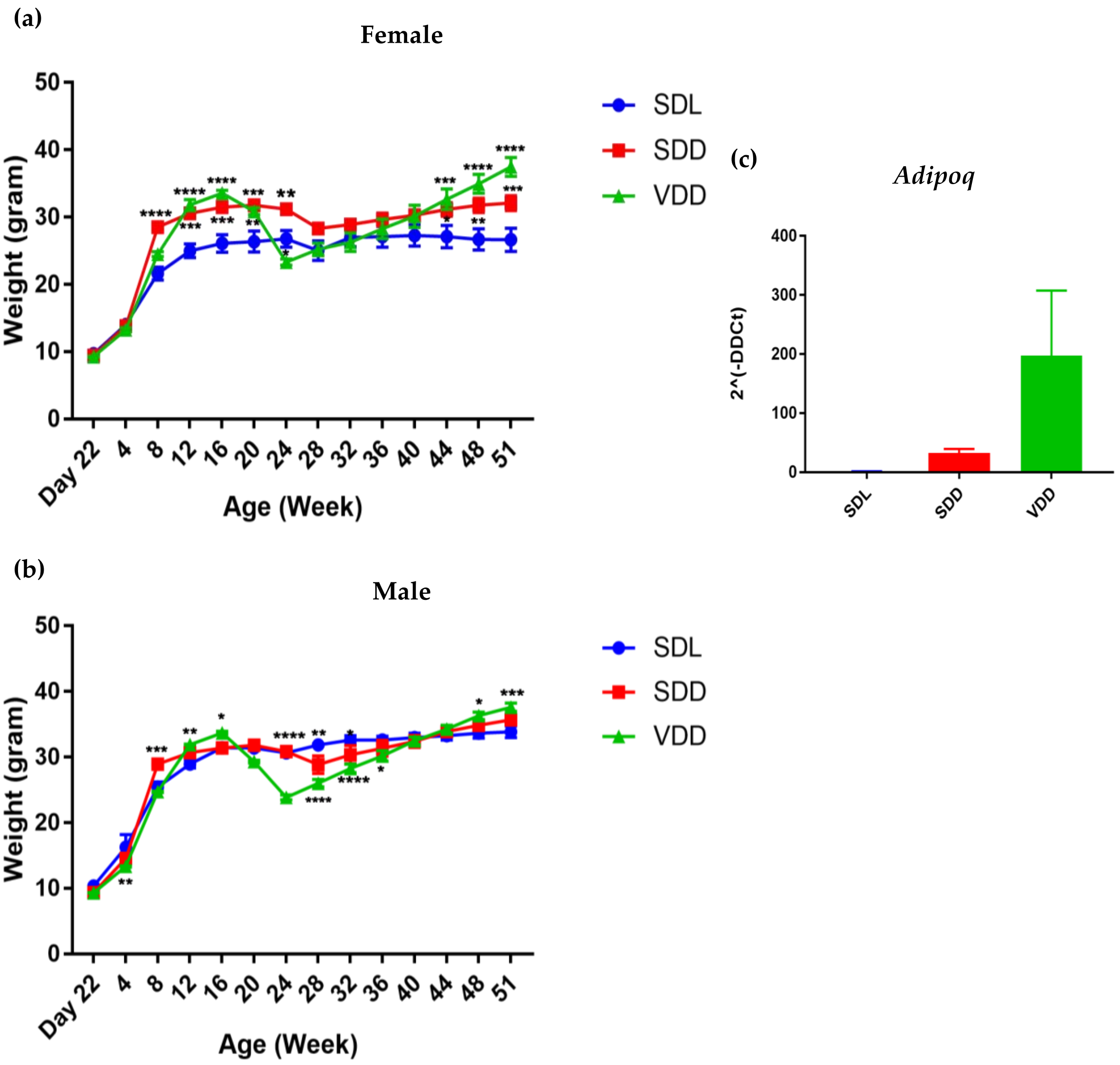

Figure S2. Changes in body weights of mice during the experimental period. (a) and (b) The three groups of mice were separated based on gender and the body weights were recorded throughout the experimental period (third week to 12 months). (c) Gene expression studies for the fat marker *Adipoq*. All mice were 12 months old, n = 8–12 per group.

Data are presented as mean ± SE. One-way ANOVA was used for data analysis. * indicates significant differences from the control group. * $p \leq 0.05$, ** $p \leq 0.01$, *** $p \leq 0.001$, **** $p \leq 0.0001$

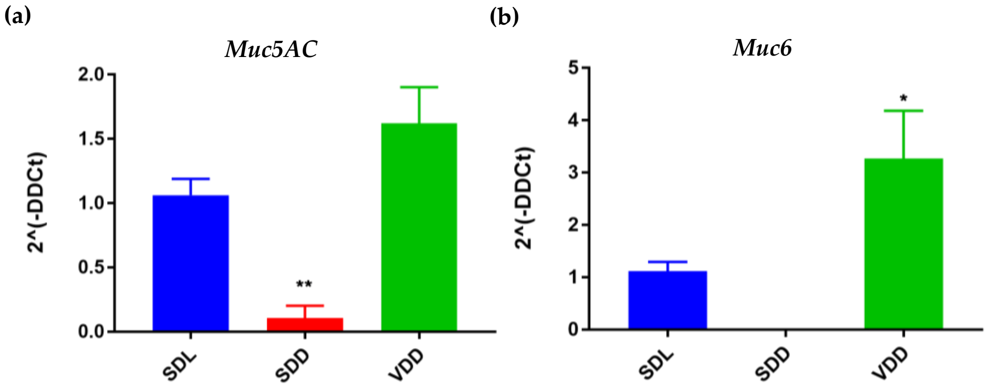

Figure S3. Gene expression analysis of (a) *Muc5AC* and (b) *Muc6* by qRT-PCR. The mice were 12 months old, n = 8–12 per group. Data are presented as mean ± SE. One-way ANOVA was used for data analysis. * Indicates significant differences from the control group. *$p \leq 0.05$, **$p \leq 0.01$.

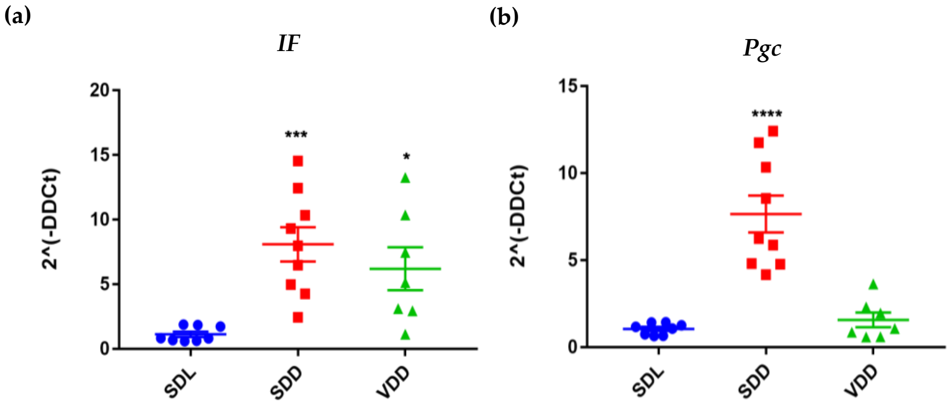

Figure S4. VD deficiency and zymogenic cells. Gene expression studies (2^(-DDCt)) for a) *IF* and b) *Pgc.* Data are presented as mean ± SE. One-way ANOVA was used for data analysis. The mice were 12 months old, n = 7–9 per group). * indicates significant differences from the control group. *$p < 0.05$, ***$p \leq 0.001$, ****$p \leq 0.0001$.

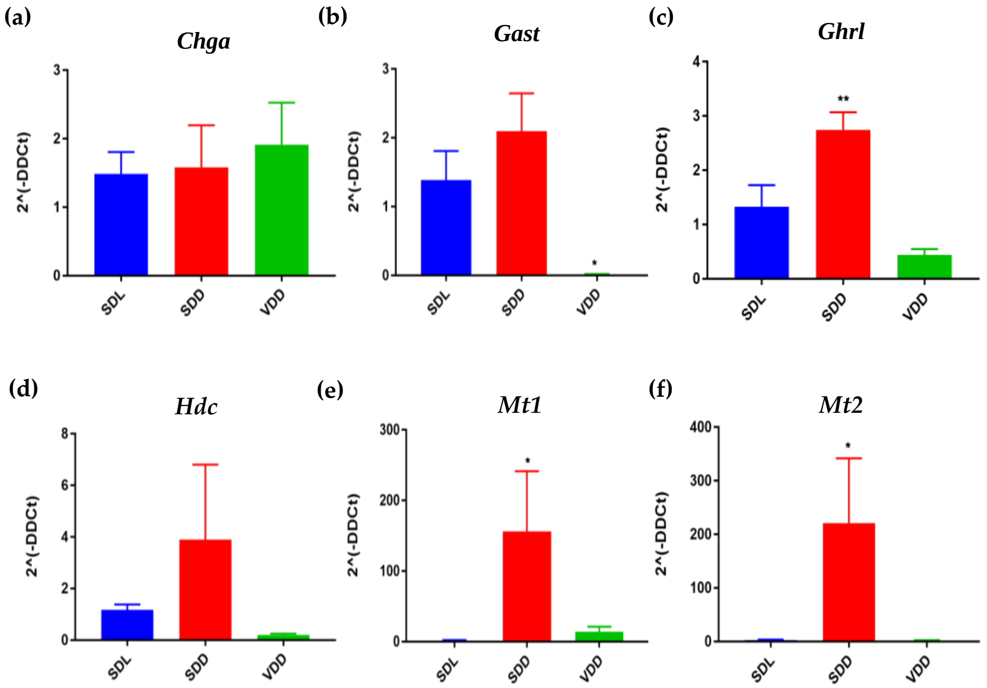

Figure S5. VD deficiency affects the gene expression of hormones. (a) *Chga*; (b) *Gast*; (c) *Ghrl*; (d) *Hdc*; (e) *Mt1* and (f) *Mt2* expression levels were determined by qRT-PCR. All mice were 12 months old, n = 7–12 per group. Results are presented as mean ± SE. One-way ANOVA was used for data analysis. * indicates significant differences from the control group. *$p \leq 0.05$, **$p \leq 0.01$.

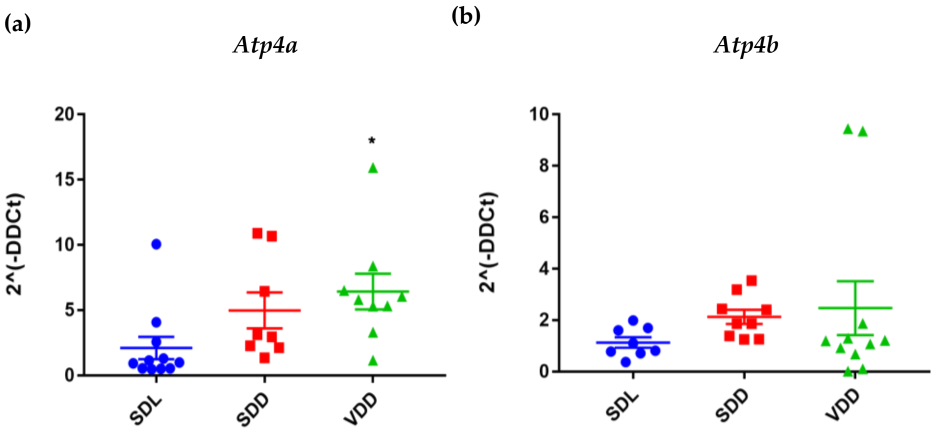

Figure S6. VD deficiency and parietal cell abnormal function. Analysis of (a) *Atp4a* and (b) *Atp4b* in the mouse gastric tissues as revealed by qRT-PCR (2^(-DDCt)); (d) Acid content measurement. Data are presented as mean ± SE. All data were obtained from 12-month-old mice, n = 8–11 per group. * indicates significant differences from the control group. * $p < 0.05$.

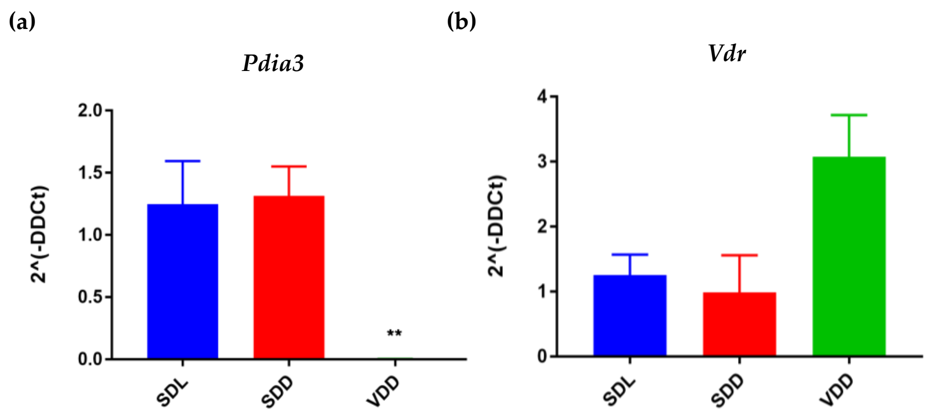

Figure S7. Distribution of VD receptors in the stomach. a) *Pdia3* and (b) *VDR* gene expression levels (2^(-DDCt)) as determined by qRT-PCR and normalized to *GAPDH*. All mice were 12 months old, n = 8–12 per group. Data are presented as mean ± SE. One-way ANOVA (Dunnet's test) was used for data analysis. * indicates significant differences from the control group. ** $p \leq 0.01$.

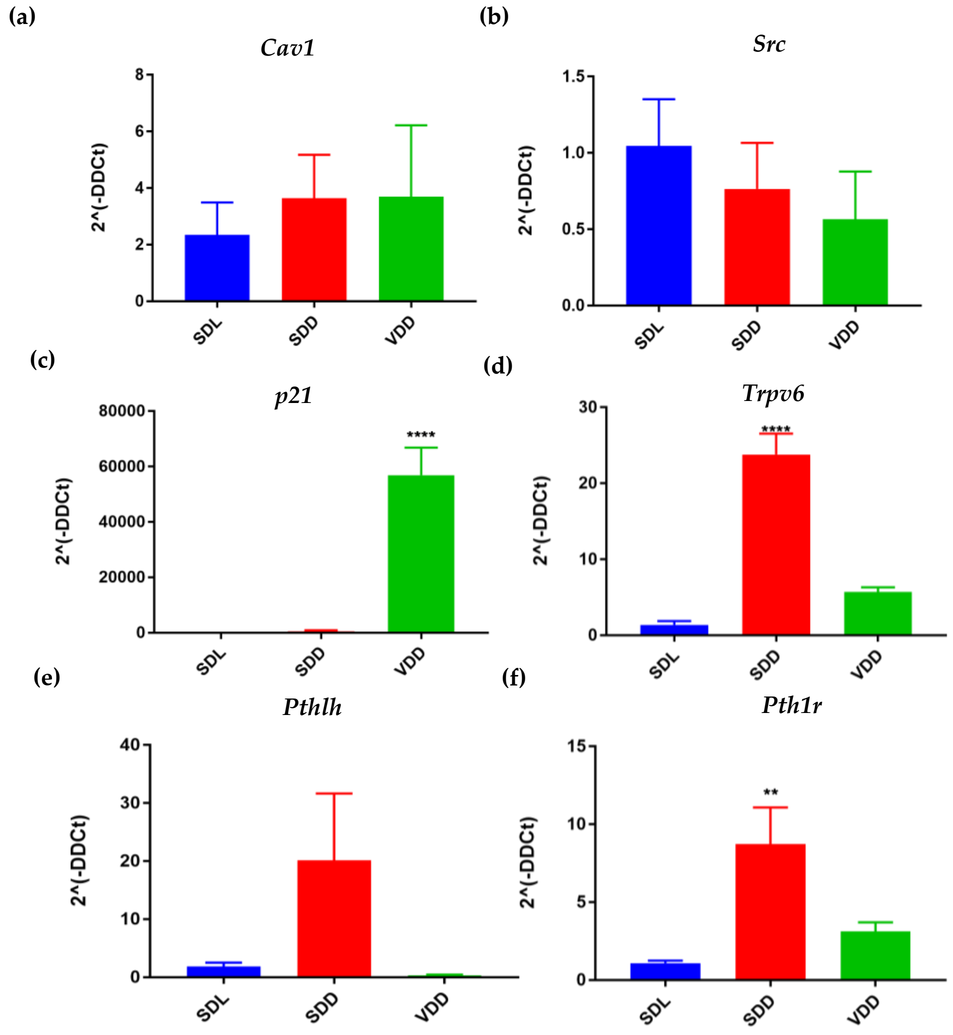

Figure S8. nVDR and Pdia3 target gene expression in VD deficient mice (a) *Cav1* and (b) *Src*, (c) *p21*; (d) *Trpv6*; (e) *Pthlh*; and (f) *Pth1r*. The mice were 12 months old (n = 8–11 per group). Data are presented as mean ± SE, One-way ANOVA was used for data analysis. * indicates significant differences from the control group. ** $p \leq 0.01$, **** $p \leq 0.0001$
